# An Updated Review of Ciguatera Fish Poisoning: Clinical, Epidemiological, Environmental, and Public Health Management

**DOI:** 10.3390/md15030072

**Published:** 2017-03-14

**Authors:** Melissa A. Friedman, Mercedes Fernandez, Lorraine C. Backer, Robert W. Dickey, Jeffrey Bernstein, Kathleen Schrank, Steven Kibler, Wendy Stephan, Matthew O. Gribble, Paul Bienfang, Robert E. Bowen, Stacey Degrasse, Harold A. Flores Quintana, Christopher R. Loeffler, Richard Weisman, Donna Blythe, Elisa Berdalet, Ram Ayyar, Danielle Clarkson-Townsend, Karen Swajian, Ronald Benner, Tom Brewer, Lora E. Fleming

**Affiliations:** 1 Mount Sinai Medical Center, Miami Beach, FL 33140, USA; 2 Department of Psychology and Neuroscience, Nova Southeastern University, Fort Lauderdale, FL 33314, USA; mf934@nova.edu; 3 National Center for Environmental Health, Centers for Disease Control and Prevention, Atlanta, GA 30341, USA; lfb9@cdc.gov; 4 Marine Science Institute, University of Texas at Austin, Port Aransas, TX 78373, USA; robt.dickey@austin.utexas.edu; 5 Jackson Memorial Medical Center, Department of Medicine, University of Miami, Miami, FL 33136, USA; jbernstein@med.miami.edu (J.B.); kschrank@med.miami.edu (K.S.); 6 Florida Poison Information Center, University of Miami Miller School of Medicine, Miami, FL 33136, USA; wstephan@med.miami.edu (W.S.); rweisman@med.miami.edu (R.W.); 7 NOAA-National Ocean Service, Center for Coastal Fisheries and Habitat Research, Beaufort, NC 28516, USA; steve.kibler@noaa.gov; 8 Department of Environmental Health, Emory University Rollins School of Public Health, Atlanta, GA 30322, USA; matt.gribble@emory.edu (M.O.G.); danielle.clarkson-townsend@emory.edu (D.C.-T.); 9 Oceanography Department, University of Hawaii, Honolulu, HI 96822, USA; bienfang@soest.Hawaii.edu; 10 School for the Environment, University of Massachusetts Boston, Boston, MA 02125, USA; bob.bowen@umb.edu; 11 Food and Drug Administration, College Park, MD 20740, USA; stacey.degrasse@fda.hhs.gov (S.D.); karen.swajian@fda.hhs.gov (K.S.); 12 Food and Drug Administration, Dauphin Island, AL 36528, USA; harold.floresquintana@fda.hhs.gov (H.A.F.Q.); christopher.loeffler@fda.hhs.gov (C.R.L.); ronald.benner@fda.hhs.gov (R.B.); 13 Private Practice, Miami, FL 33133, USA; blythedrdiva@aol.com; 14 Institut de Ciències del Mar (CSIC), 08003 Barcelona, Spain; berdalet@icm.csic.es; 15 Department of Neurology, University of Miami, Miami, FL 33136, USA; dayyar@med.miami.edu; 16 Northern Institute, Charles Darwin University, Darwin 0909, Australia and Australian Institute of Marine Science, Darwin 0811, Australia; tom.brewer@cdu.edu.au; 17 European Centre for Environment and Human Health, University of Exeter Medical School, Truro, Cornwall Tr1 3HD, UK; l.e.fleming@exeter.ac.uk

**Keywords:** ciguatera fish poisoning, ciguatoxin, harmful algal bloom, algae, seafood poisoning, *Gambierdiscus*, diagnosis, treatment, human health, natural toxins, climate change, environmental change, food poisoning, neuropsychology, neurology, neurotoxin

## Abstract

Ciguatera Fish Poisoning (CFP) is the most frequently reported seafood-toxin illness in the world. It causes substantial human health, social, and economic impacts. The illness produces a complex array of gastrointestinal, neurological and neuropsychological, and cardiovascular symptoms, which may last days, weeks, or months. This paper is a general review of CFP including the human health effects of exposure to ciguatoxins (CTXs), diagnosis, human pathophysiology of CFP, treatment, detection of CTXs in fish, epidemiology of the illness, global dimensions, prevention, future directions, and recommendations for clinicians and patients. It updates and expands upon the previous review of CFP published by Friedman et al. (2008) and addresses new insights and relevant emerging global themes such as climate and environmental change, international market issues, and socioeconomic impacts of CFP. It also provides a proposed universal case definition for CFP designed to account for the variability in symptom presentation across different geographic regions. Information that is important but unchanged since the previous review has been reiterated. This article is intended for a broad audience, including resource and fishery managers, commercial and recreational fishers, public health officials, medical professionals, and other interested parties.

## 1. Introduction

Ciguatera Fish Poisoning (CFP) is the most commonly reported natural marine toxin related illness globally [1]. It is characterized by gastrointestinal, neurologic, and cardiac symptoms. Humans contract CFP by eating finfish containing the naturally occurring toxins, ciguatoxins (CTXs). CFP is not due to the mishandling of fish and is not prevented by any particular storage, preparation, or cooking methods. The CTXs are tasteless, colorless, odorless, heat and acid stable, and stable for at least six months at commercial freezing temperatures [2,3]. Thus, prevention and management of CFP require a multidisciplinary approach.

CTXs and their precursors are produced by microscopic algae known as dinoflagellates, in the genus, *Gambierdiscus.* These dinoflagellates are bottom-dwelling [4] and are typically found attached to seaweeds, living and dead corals, and other substrates (i.e., surfaces) in shallow tropical and subtropical waters. Due to *Gambierdiscus*’ requirements for light and substrate, CTX production occurs in shallow coastal habitats (e.g., reefs, atolls). Oceanic fish whose nutrition is derived from pelagic (i.e., in open sea waters and away from the shore) food webs rather than shallow/coastal food webs are less susceptible to the accumulation of CTXs. Table 1 provides examples of fish commonly associated with CFP.

CTXs are transferred and metabolized through the food web, as *Gambierdiscus* cells are ingested by herbivorous fish, which are then consumed by piscivorous fish, both of which are then consumed by humans. It is believed that CTXs are bioaccumulated and concentrated, such that fish higher in the food web tend to contain the highest CTX concentrations [16,17,18,19,20]. However, recent studies from French Polynesia and Hawaii indicate a general lack of relationship between fish size and ciguatera toxicity for many species and families of fish, suggesting that fish size alone is not an adequate predictor of toxicity [21,22] at least in those regions.

While *Gambierdiscus* is known to produce two main types of toxins, the water-soluble maitotoxins and the lipid-soluble CTXs, maitotoxins have no proven role in CFP [23,24]. Examples of the CTX structures from *Gambierdiscus* spp. and their closely related isomers and congeners, isolated from fish collected in the Pacific Ocean [25,26], Caribbean Sea [2,27] and Indian Ocean [28], are summarized in Yasumoto and Murata (1993) [29] as well as Dickey (2008) [30], and the references therein.

Historically, CFP was associated with isolated island communities that relied heavily on subsistence fishing for their food supply [31,32], but the geographic reach of CFP has expanded due to international seafood trade and travel [33,34,35,36,37]. In addition, people consuming fish from areas previously free of CFP may be at risk when such areas become prone to CFP [38,39,40] due to changes in the aquatic environment. It has also been speculated that CTXs may accumulate in farm-raised fish not typically associated with CFP via feed composed of wild fish contaminated with CTX [41]. Thus, the human populations that need to be incorporated into CFP risk assessments include not only those in endemic areas, but also tourists and recreational fishers traveling to endemic areas, transients (e.g., ships’ crews visiting endemic areas), and people in other regions consuming fish imported from endemic areas [36,38,42,43,44].

It is notable that the fish commonly associated with CFP are among the top selling species in many markets. In Florida in 2013, for instance, certain species of Grouper (Serranidae family), Snapper (Lutjanidae family), Mackerel (Scombridae family), and Jacks (Carangidae family) were among the top 20 types of finfish sold [45]. In 2014, 41.3 metric tons of barracuda were sold in Florida [46,47], a significant seafood market, given that barracuda is broadly viewed as ha ving an extremely high potential to be ciguatoxic. This highlights the importance of awareness on the part of the public and healthcare professionals, as well as the development of effective CFP risk management systems on a local to global scale.

## 2. Human Health Effects and Diagnosis

The diagnosis of CFP is based on the recent fish-eating history of the patient(s), clinical presentation, and whenever possible, results from analytical testing of a remnant of the fish consumed by the person(s) suffering from CFP. Multiple individuals consuming the same fish or related lot (i.e., catch) of fish, with several individuals experiencing signs, symptoms, and time course consistent with CFP, strongly supports the diagnosis [48]. The diagnosis also requires that other conditions with similar presentations be considered and ruled out (see Differential Diagnosis section). A number of case definitions or descriptions have been used for CFP [49,50]. A universal case definition, such as the example in Box 1 could help identify cases consistently.

Box 1 **Possible universal case definition of ciguatera fish poisoning (CFP).**

**A case definition is a set of uniform criteria for identifying a disease, which is used for research purposes, clinical diagnosis, or public health surveillance. With regard to CFP, a universal case definition, designed to account for the variability in symptom presentation for fish obtained from different geographic regions (e.g., Caribbean Sea, Indian Ocean and Pacific Ocean), is desirable to help identify cases consistently. Following is a possible case definition. This proposed definition is a refinement or modification of other CFP clinical descriptions or case definitions (e.g., Centers for Disease Control and Prevention’s Yellow Book [49], US Food and Drug Administration’s Bad Bug Book [51], European Food Safety Authority’s Framework Agreement [50]), for global application:**

Clinical Criteria:

Patient who consumed a saltwater (marine) fish that has been previously associated with CFP, AND, reports neurologic symptoms which may include any combination and sequence of paresthesia, dysesthesia, pruritus, allodynia, myalgia, arthralgia, and dizziness with onset up to approximately 48 h after eating the fish.

Gastrointestinal (GI) symptoms (e.g., nausea, vomiting, diarrhea) often precede or accompany the neurological symptoms, with GI symptom onset usually within minutes to 12 h after fish consumption. Cardiovascular symptoms and signs (hypotension and bradycardia) may also be present.

Laboratory Criteria:

Confirmation of ciguatoxin(s) in implicated raw or cooked fish meal remnant.

Epidemiological Criteria:

Exposure to the same fish source as a confirmed CFP case.

Case Classification:

· Confirmed case: Any patient meeting the clinical and laboratory criteria.
· Probable case: Any patient meeting the clinical and epidemiological criteria.
· Possible case: Any patient meeting the clinical criteria after consuming a saltwater (marine) fish that is either NOT previously associated with CFP or of unknown species; or any patient with an illness presentation that differs slightly from the clinical criteria or is an unusual presentation that, in the professional judgment of the healthcare provider, merits consideration for a CFP diagnosis; or any patient who meets the clinical criteria but where other etiologies have not been ruled out.

Outbreak definition:

Two or more cases that are epidemiologically related.

### 2.1. Signs and Symptoms

CFP is characterized by gastrointestinal, neurological, and cardiovascular symptoms. In addition, after the initial or acute illness, neuropsychological symptoms may be reported. Clinical features can vary depending on elapsed time since eating the toxic meal, and whether the geographic source of the implicated fish was the Caribbean Sea, Pacific, or Indian Ocean [17,36,52,53,54,55,56,57,58].

Table 2 summarizes the reported frequency of CFP symptoms in a variety of studies.

Gastrointestinal symptoms and signs usually begin within 6–12 h of fish consumption and resolve spontaneously within 1–4 days. Gastrointestinal symptoms may include nausea, vomiting, abdominal pain, and diarrhea.

The neurologic symptoms usually present within the first two days of illness. They often become prominent after the gastrointestinal symptoms (particularly in CFP events from Caribbean fish), although they may present concurrently with gastrointestinal symptoms (K. Schrank, written communication, April 2016) [59]. The neurologic symptoms vary among patients and include paresthesias (i.e., numbness or tingling) in the hands and feet or oral region, metallic taste, sensation of loose teeth, generalized pruritus (itching), myalgia (muscle pain), arthralgia (joint pain), headache, and dizziness. A distinctive neurologic symptom is cold allodynia, sometimes referred to as “hot–cold reversal,” an alteration of temperature perception in which touching cold surfaces produces a burning sensation or a dysesthesia (i.e., unpleasant, abnormal sensation) [60]. One study revealed that intra-cutaneous injection of CTX in humans elicited this sensation [61]. Cold allodynia is considered pathognomonic of CFP, although not all patients report experiencing it (see Table 2) and it can be seen with other human seafood poisoning syndromes (e.g., neurotoxic shellfish poisoning). Less commonly, severe central nervous system symptoms, such as coma or hallucinations, have been reported [54,62,63].

Neuropsychological symptoms, which often become apparent in the days or weeks after the initial or acute illness, include subjectively reported cognitive complaints such as confusion, reduced memory, and difficulty concentrating [64,65,66,67], depression or irritability [64,65,68], and anxiety [65]. Fatigue or malaise have been reported and may be debilitating [6,62,69,70].

Cardiac symptoms and signs may manifest, generally in the early stage of the illness. When present, they usually occur in combination with gastrointestinal and/or neurologic signs and symptoms [71,72]. Cardiac signs often include hypotension and bradycardia which may necessitate emergency medical care.

Diagnosing CFP requires careful attention to the time course of symptoms. Patients who present for healthcare early in the illness, when gastrointestinal symptoms are salient, may be difficult to differentiate from patients with other types of food poisoning. Patients presenting with bradycardia or neurologic symptoms after gastrointestinal symptoms have resolved, may not recall, or may minimize, previous gastrointestinal symptoms. Therefore, healthcare providers who are considering CFP as part of a differential diagnosis should inquire about symptoms experienced earlier in the course of illness.

Geographic differences in symptoms and progression have been described and may be attributed to the presence of different CTX congeners (i.e., chemical structure variants) in different geographic regions [81]. In the Caribbean, gastrointestinal symptoms and signs predominate in the acute phase (i.e., first 12 h) [75], with the subsequent prominence of neurologic, especially peripheral neurologic symptoms (Box 2). In the Pacific, neurological symptoms and signs may predominate acutely [52] and there have been a few reports of more severe acute neurologic effects including coma [54]. In the Indian Ocean, CFP has been associated with neurological, neuropsychiatric, and mental status alterations, with reports of hallucinations, giddiness, incoordination, and loss of equilibrium [62].

Box 2 **A classic case of CFP from Caribbean fish.**

**CFP symptom presentation varies by geographic origin of the consumed fish. For fish originating from the Caribbean, the following is an example of a typical case:**

A 50-year-old man presented with nausea, vomiting, and diarrhea, followed within 24 h by itchy skin without a rash and a burning sensation when touching cold objects. He and his wife returned from the Bahamas 2 days ago, where they had caught and eaten a large grouper which did not taste bad or spoiled, within hours prior to the onset of symptoms. His wife was complaining of similar symptoms [82].

CFP is rarely fatal [83]. Death may occur in severe cases due to dehydration, cardiovascular shock, or (very rarely) respiratory failure resulting from paralysis of the respiratory musculature [55,84], especially in areas where ventilatory support and emergency medical care are unavailable. When fatal, the illness is often characterized by convulsions, coma, and focal neurological deficits (see Chan, 2016 [83], for review). Anecdotally, in some of these authors’ experiences primarily with Caribbean cases, critical complications are generally readily apparent upon initial clinical evaluation (Written communication, K. Schrank, J. Bernstein, D. Blythe, L.E. Fleming, September 2016) [85]. Consumption of the fish head or organs (e.g., liver or gonads) is associated with greater symptom severity than eating only the fillet, as CTXs accumulate in greater concentrations in these organs [86,87].

### 2.2. Other Features

There are isolated reports suggesting transmission of CFP from a pregnant mother with CFP to the fetus [88], and from a nursing mother with CFP to an infant [89]. There have also been reports of tingling and localized paresthesias in the sexual partners of CFP patients during the acute phase of the illness [90,91].

As part of a larger study, Gatti et al. (2008) [77] described the case of a woman, 32 weeks pregnant, who became ill with a CFP-like intoxication subsequent to consuming fish. The patient was hospitalized and treated (corticosteroid, antipruritic, and anti-emetics) with no apparent impact on the fetus at the time. This report provided no additional information about whether there were subsequent complications for the mother or the fetus. Documented clinical experience with CFP during pregnancy is sparse.

### 2.3. Differential Diagnosis

CFP induces some symptoms in common with paralytic shellfish poisoning (PSP), neurotoxic shellfish poisoning (NSP), scombrotoxin fish poisoning, pufferfish poisoning (also referred to as Fugu poisoning), and hallucinatory fish poisoning [92]. Other maladies with similar symptomology include botulism, enterovirus infection [93], bacteremia [94], organophosphate pesticide poisoning, severe electrolyte disturbances from extreme vomiting and diarrhea, eosinophilic meningitis, multiple sclerosis, Guillain–Barre Syndrome, and other neurologic conditions [77].

Cold allodynia may be experienced by patients suffering from either NSP or CFP. NSP is caused by human consumption of shellfish contaminated by brevetoxins, a group of compounds produced by marine dinoflagellates in the genus *Karenia* [95] which are similar structurally to CTXs. Although brevetoxins can accumulate in muscles and viscera of finfish [96], there are as yet no documented cases of NSP after finfish consumption in humans. Therefore, in presumptive CFP patients presenting with temperature dysesthesia, a thorough history of recent seafood consumption should be obtained. If the recent consumption history identifies shellfish and not finfish, then the differential diagnosis of NSP is more likely.

Gastrointestinal and neurologic symptoms such as paresthesias may indicate CFP; however, sphincter dysfunction, optic neuritis, or internuclear ophthalmoplegia point to central nervous system involvement, as seen in multiple sclerosis. Also, symptoms of CFP can be confounded with psychological or psychosomatic conditions [97]. Table 3 provides some examples of illnesses with clinical features in common with CFP.

### 2.4. Symptom Duration

After the initial or acute illness, many symptoms may persist a few days to several weeks or months [44,72,78,100], including, peripheral neurologic symptoms (e.g., paresthesias in the extremities and pruritus), and systemic symptoms (e.g., malaise, weakness, generalized fatigue, and headaches).

Neuropsychological symptoms (e.g., depression, anxiety, inability to concentrate, subjective memory loss) [7,64,65,68,78,86,101,102,103,104] have also been reported, after the initial or acute illness. One longitudinal study assessed symptoms in 12 CFP patients and 12 healthy control subjects, at a baseline evaluation (within a few weeks after intoxication) and at a six-month follow-up evaluation. At the baseline evaluation, CFP cases endorsed significantly greater symptoms on a questionnaire assessing perceived symptom severity (e.g., fatigue, muscle weakness, tingling sensations/paresthesias, confusion, word-finding difficulty, concentration problems, irritability) and greater anxiety symptoms than controls. Follow-up evaluations revealed resolution of all symptoms after six months. Of note, almost all of the CFP cases in that study were poisoned by fish from Caribbean waters, and the chronicity of symptoms in Caribbean cases may differ from that of Pacific or Indian Ocean cases [52,65].

Although rare, there are reports of CFP symptoms lasting for years in some patients [7,75]. However, further research is needed to rule out the potentially confounding influence of other medical or psychological conditions, and to substantiate such a long-term course of illness.

### 2.5. Symptom Recurrence

Frequent anecdotal reports to physicians and investigators indicate that after experiencing CFP, ingesting alcohol [6,34,80,81,88,105,106,107] or any fish (including freshwater species) [6,80,81,88,105] can cause a recurrence of symptoms within the weeks or months following initial poisoning. Ingesting caffeine [88], nuts [88,108], chicken [6,108] and pork [108], or experiencing physical over-exertion or dehydration [34] have also been reported anecdotally to be associated with symptom recurrence. Such recurrence may reflect a cumulative exposure process, neurological sensitization [109,110], cross-sensitization, or metabolic remobilization of CTX from fatty tissue [52]. See Table 4 for a list of items associated with symptom recurrence.

When symptoms recur years after the initial exposure [106,111], alternative etiologies for symptoms should be ruled out, as presumed CFP symptoms and signs may actually be indicators of other pathology [94].

### 2.6. Biomarkers

There are currently no identified biomarkers that can be used to confirm exposure to CTX in humans [112,113,114,115,116]. The current “gold standard” diagnosis of CFP includes the detection of CTXs in the implicated fish by appropriate analytical testing methods (see CTX Detection in Fish, below), in addition to a history, clinical manifestation, and time course consistent with CFP.

## 3. Pathophysiology of CFP

Ciguatoxins have been shown to bind to voltage-gated sodium channels [117]. At the resting membrane potential of axons, sodium channels are closed. CTX molecules induce the opening of these channels at resting membrane potential, leading to an influx of sodium, a process that depolarizes the axonal membrane and triggers spontaneous and repetitive action potentials (AP). Normally, gated influx of sodium is accompanied by an efflux of potassium, which maintains electroneutrality within the axon and mediates the water movement across the membrane [118]. When the dynamic process of influx/efflux is interrupted due to CTX, it results in swelling at the nodes of Ranvier (the unmyelinated sections of axons exposed to extracellular fluid in myelinated axons) [119]. The swelling of the nodes of Ranvier impairs the saltatory conduction along the axon and slows sensorimotor conduction velocities [101,120].

Animal laboratory research shows that CTXs are rapidly absorbed from the gastrointestinal tract and distributed throughout the body [116]. Moreover, because CTX-sensitive voltage-gated sodium channels have been found in all of the systems affected by CFP (i.e., brain, skeletal muscle, heart, peripheral nervous system, sensory neurons), these channels may mediate the symptomatology of CFP [121,122,123,124,125,126,127,128,129,130,131,132,133]. As an example, cold allodynia may be attributable to a CTX-induced modification of the voltage-gated sodium channels in A-delta and C fibers [134], which carry thermal and pain impulses through the spinal cord to the brain [135]. Of note, nerve conduction studies of patients with acute CFP reveal a disturbance in both sensory and motor conduction [101].

With regard to the central nervous system, one animal study showed that CTX administered orally and intraperitoneally was detected four days after exposure in the liver, muscle, and brain [116], suggesting that CTX crosses the blood–brain barrier. This supports the observation of central nervous system involvement in human clinical cases of CFP [77,136,137,138].

## 4. Treatment

Over the years, many treatments have been tried, mostly involving symptomatic and supportive care. This section describes treatments that have been used during and after the acute stage. Intravenous (IV) mannitol is the only treatment whose effectiveness specifically for CFP has been investigated by randomized clinical trials.

### 4.1. Acute Symptomatic and Supportive Treatments

With acute CFP, cardiorespiratory support and the correction of acid–base or electrolyte disturbance is of utmost importance [81]. As with any acute poisoning associated with volume depletion, IV fluid resuscitation with large volumes of isotonic fluids may be necessary for patients in shock, with the addition of an IV pressor infusion if needed after volume repletion. Symptomatic bradycardia may require IV atropine dosing as needed (0.5 mg every 3 to 5 min to maintain a heart rate goal of 60 beats per minute, with no maximum total dosage limit), and/or temporary cardiac pacing to achieve hemodynamic stability. Refractory bradycardia with hypotension may respond to a controlled infusion of chronotropic pressors (e.g., epinephrine). Rarely, critically ill CFP patients may require endotracheal intubation and mechanical ventilation for either airway protection and/or for respiratory failure. The prognosis for full recovery of critically ill patients is excellent with appropriate intensive care treatment when needed. Patients seen within the first few hours after ingestion of toxic fish may benefit from treatment with oral activated charcoal to prevent further absorption of toxin from the gut, although severe vomiting associated with CFP may preclude its administration [108,139].

Various medical treatments for other CFP symptoms have been tried with variable success [131], and randomized controlled trials to establish their effectiveness are lacking. Acutely, ondansetron (8 mg IV) or other anti-emetics may reduce nausea and vomiting. Acetaminophen and nonsteroidal anti-inflammatory medications may be needed for initial analgesia. Caution is warranted in prescribing opiates and barbiturates since they may lead to hypotension, and because opiates may interact with maitotoxins, natural marine toxins that may also be present in ciguatoxic fish even though they are not known to play a role in CFP [88].

During the first 1–2 h after ingestion, it is generally accepted that vomiting may aid the body in toxin elimination, but later prolonged vomiting would add little benefit after the initial emptying of the stomach. Since diarrhea may also aid in the elimination of the toxin, it is generally not suppressed, but suppression may become necessary to prevent excessive fluid loss. Monitoring of hydration and electrolyte balance is essential.

### 4.2. Mannitol

Intravenous mannitol remains the primary treatment consideration for CFP. IV mannitol infusion is the most studied therapy for CFP, and the only therapy evaluated by randomized, blinded clinical trials [79,140]. Mannitol therapy has been recommended for the goal of reducing symptoms (especially neurologic) during the acute stage of the illness, and for shortening the duration of symptoms after the acute stage. Because it is an osmotic diuretic, it should be given following the restoration of adequate intravascular volume with isotonic intravenous fluids; and throughout mannitol treatment, the hydration and electrolyte status should be carefully monitored [141].

IV mannitol is administered at 1.0 g/kg body weight over a 30–45 min period [93] although there is some variability in different reports (e.g., dosage 0.5 to 1 g/kg, and administration duration up to 3–4 h) [7,142,143]. It is suggested that mannitol be given within 48–82 h of ingestion of the suspect fish [143,144] for optimal benefit, although beneficial effects have been reported up to several weeks after initial poisoning. Repeated treatments may be needed, especially if there is a positive but partial response to treatment [7,145].

Some of the risks of mannitol treatment include the loss of additional fluids and electrolytes in patients suffering from acute diarrhea and vomiting; and patients experiencing bradycardia and hypotension may be at higher risk of cardiac failure if infused with high doses of mannitol [52]. Therefore, frequent clinical reassessments are needed to guide interventions. Additionally, mannitol can be caustic and toxic to tissues, causing burning on administration and tissue damage in the event of extravasation. A large bore IV in a large vein (such as the antecubital) is recommended both to prevent extravasation and for the comfort of the patient.

The effect of mannitol is thought to be mediated by the reduction of neuronal edema [68] through the modulation of sodium ion concentrations across cell membranes. In animals, mannitol reverses membrane excitability and nodal swelling caused by CTX [146,147]; and it is thought to have similar effects in humans [136]. Also, mannitol may act as a scavenger of free radicals generated by the CTX molecule and may reduce the action of CTX at the sodium and/or potassium channels [148].

Research on the benefits of mannitol consists of a number of descriptive reports, e.g., [93,143,145] and two randomized trials [79,140]. Mannitol has been associated with significant, rapid improvement, and even resolution of acute CFP signs and symptoms [93,143,145]. Anecdotal reports suggest that mannitol treatment is associated with the reduced likelihood of returning for additional medical care [7]. One randomized clinical trial found that mannitol was associated with a significantly greater reduction in symptoms than a non-mannitol treatment [140]. However, a double-blind randomized trial [79], did not find mannitol to be superior to a comparison treatment of normal saline (see Friedman et al., 2008 [1] for review and discussion of study design issues).

### 4.3. Treatment after the Acute Illness

Beyond the acute period post ingestion, most neurologic and constitutional symptoms are relatively mild, self-limited, and not severe enough to warrant chronic pharmacologic intervention. Nevertheless, medications that have been used include fluoxetine for ongoing fatigue [149]; amitriptyline for paresthesias, pruritus, and headaches [34,150]; and paracetamol (acetaminophen) and nifedipine for headaches [57,149]. Gabapentin has been used to treat chronic pain [151]; however, caution is warranted when prescribing medications with addiction potential given that there are no randomized controlled trials examining their safety and effectiveness for the treatment of CFP. There may be a benefit of using pregabalin (LYRICA^®^, Pfizer) for painful dysesthesia [152]. There was one case report describing a beneficial effect of hypnosis to remediate symptoms in a presumed CFP patient [153].

Supportive discussions and anticipatory education about the expected illness course with each patient and their family is an important component of post-acute care. In particular, it may be helpful to notify patients that elevated anxiety is a common feature of the ciguatera symptomatology during the weeks after initial intoxication. Such anxiety is correlated with the level of subjectively experienced physical and neuropsychological symptoms, and is expected to resolve when the other CFP symptoms resolve [65]. Such anxiety may be attributed to the patients’ psychological reaction to the novelty or strangeness of the CFP symptoms, the fact that the symptoms resemble more grave neurological conditions, and the persisting nature of the symptoms during the weeks after the initial illness [65]. Perhaps the most important element of post-acute treatment is providing reassurance and conveying the need for patience with the illness course, as most individuals see their symptoms diminish gradually and resolve completely within weeks or months. Trials with cognitive behavioral therapy or biofeedback training have not yet been done, but referral for such interventions may be appropriate and helpful.

Traditional herbal medicines and remedies have also been used to treat CFP [154]. For instance, in New Caledonia in the Pacific, at least 90 different plant species have been reportedly used including *Heliotropium foertherianum* Diane and Hilger (Boraginaceae), *Euphorbia hirta* L. (Euphorbiaceae), and *Vitex* L. sp. (Lamiaceae) [154,155]. However, adequate scientific evidence for their safety and effectiveness is still lacking.

### 4.4. Avoiding Recurrence

Patient education regarding foods and activity associated with symptom recurrence (see Table 4) is prudent, but caution is warranted due to the possibility of symptom suggestibility, i.e., the possibility that patient hypervigilance about possible symptom recurrence leads to perceived symptomatology in the absence of active disease. Again, it may be helpful to inform patients that anxiety about the symptoms is part of the CFP symptomatology, and will resolve as the other symptoms also resolve.

Patients may consider avoiding such food and activities for 3–6 months after poisoning or until all CFP-related symptoms have resolved. Patients may choose to cautiously experiment or reintroduce such foods or beverages. However, controlled investigations of the incidence of symptom recurrence, as well as the effectiveness of specific avoidance measures, have not been conducted, and any beneficial impacts may vary widely among individuals.

## 5. CTX Detection in Fish

Methods have been established for detecting CTXs in fish implicated in CFP outbreaks, the results of which are used to support the clinical diagnosis of CFP. Within the United States, the Food and Drug Administration (FDA) provides CTX testing for health officials, by receiving fish meal remnants (raw or cooked) implicated in the poisoning, and analyzing them for both species identification (through DNA barcoding [156,157]) and molecular confirmation of CTXs in the implicated fish.

Some of the challenges for CTX detection and analysis in samples of fish are low concentrations (µg/kg) of toxin present in the fish that pose a human health threat, toxin integration in complex fish tissue matrix, and the diversity of potential CTX congeners found within a single sample.

The FDA’s CTX fish testing procedure is performed in an analytical laboratory setting and utilizes a two-tiered protocol involving: (1) in vitro mouse neuroblastoma (N2a) cell assay as a semi-quantitative screen for toxicity consistent with CTX mode of action; and (2) liquid chromatography tandem-mass spectrometry (LC-MS/MS) for molecular confirmation of CTX. In tier-1 of CTX analysis, fish specimens are screened for voltage-gated sodium channel-specific activating effects on cell membranes as measured by cellular death. This screening procedure [158,159] can distinguish between toxins that activate (e.g., brevetoxins, CTXs) versus block (e.g., tetrodotoxins, saxitoxins) the target voltage-gated sodium channels, as well as differentiate between the effects of sodium channel-specific toxins and those with other modes of action. Tier-2 tests for the presence of a specific CTX congener that has been identified as a regional biomarker for CTXs. In fish from the Gulf of Mexico, Caribbean, and other regions in the Atlantic, the FDA uses the congener Caribbean ciguatoxin-1 (C-CTX-1), while Pacific ciguatoxin-1 (P-CTX-1) is used for fish from the Pacific Ocean [160]. Multiple reaction monitoring in positive ion mode is the MS/MS technique employed to confirm the presence of a specific CTX congener.

Alternative methods for CTX detection in fish include receptor binding assays [28,117,125,161,162,163,164,165], various LC-MS methods [3,9,160,162,166,167,168,169,170,171], and other less frequently employed assay systems (see the review by Lewis, 2004 [172]). Folk methods for detecting CTXs in fish may have potential utility [173], but studies are needed to establish the validity of these approaches.

Simpler cost-effective procedures that can be used on a routine basis by fishers, fish vendors, or consumers are desired. However, to develop such a validated, rapid, quantitative method, large quantities of CTX reference material, which are currently unavailable, are required. To date, there is no commercially available, rapid, cost effective, fish-testing product that has been demonstrated by independent investigations to provide CTX detection in seafood with adequate reliability or accuracy [174,175,176].

## 6. Epidemiology and Epidemiological Challenges

The epidemiology of CFP involves the study of its incidence and prevalence, and the development and testing of interventions for prevention and management. Estimates of the number of people who live in or visit tropical or subtropical areas who suffer from CFP annually range from 10,000–50,000 [108,139] to 50,000–500,000 [177,178] although the true number of cases is difficult to ascertain due to under-reporting of CFP and other challenges [31,179]. The incidence rate varies by world region [88,179]. Table 5 summarizes the various published regional reports on the incidence of CFP.

The epidemiologic evidence that is available on CFP includes individual case reports [186] noting unusual presentations (e.g., transfer through breast milk [89] or sexual intercourse [90,91]), as well as outbreak or disease cluster investigations (e.g., family or clusters with a history of consumption of same contaminated fish), case series (e.g., collected reports to Poison Information Centers, hospitals, public health authorities, or individual scientist databases), and community studies [1,187,188]. Community studies, usually involving circumscribed island communities, have been important sources of localized prevalence and sometimes incidence statistics.

Our ability to assess CFP epidemiology has been limited due to a number of challenges, including: (1) lack of readily available, fast, and inexpensive methods to detect CTXs in fish; (2) lack of biomarkers to confirm the diagnosis in humans; (3) lack of knowledge about the illness among healthcare professionals; (4) lack of reporting of CFP to public health and other authorities; and (5) difficulty diagnosing CFP due to the non-specific nature of its (often subjective) symptoms, geographic differences in illness presentation, and variability in the severity and duration of symptoms among patients within regions. In addition, unless the diagnosis was confirmed by testing the meal remnants, it is unknown whether the case reports or the participants in clinical trials of CFP treatments were true cases of CFP. These factors lead to the misdiagnosis and misclassification of CFP, both clinically and in public health statistics, resulting in lost treatment opportunities and possible underestimates of the prevalence and incidence of CFP [1,182,187,188,189].

Internationally, it is estimated that only 10%–20% of CFP cases are formally reported to public health and other authorities; therefore, national statistics are assumed to be grossly underestimated [190]. In the United States, a statistical model using data from the Centers for Disease Control and Prevention (CDC) and from poison control centers revealed that for every reported CFP outbreak, there are an estimated 259 unreported cases [188]. In Florida, which has CFP-endemic areas, it was estimated that only 7% of diagnosed CFP cases are reported to the State Department of Health [182].

Under-reporting occurs in part because individuals with CFP often do not seek medical attention, particularly in locations where CFP is well-known to local residents. However, even if medical attention is sought, health professionals may not recognize the illness or report it to health authorities. In one study [82], a classic case of Caribbean CFP (see Box 2) was presented to 36 physicians in South Florida, where CFP is endemic. While 68% of the physicians diagnosed CFP correctly, only ~47% of those were aware that CFP was a reportable disease in Florida (i.e., required by law to be reported to the local or state health department). Also, there is often limited access to fish testing for CTX confirmation, particularly in developing nations.

Another factor contributing to under-reporting is whether or not a given case is part of an outbreak. A study of CFP in South Florida, for instance, revealed that potential CFP cases were less likely to be reported or confirmed when a given case was a single illness, as compared to being part of an outbreak or cluster of CFP cases [187].

CFP case definitions found in the existing epidemiological literature and official public health reporting guidelines are inconsistent and vary widely. For instance, some case definitions require only self-diagnosis or self-report [76,182], others require diagnosis by a physician [71], and others require testing of a remnant of the consumed fish to confirm CFP [104,191]. There is also variability in the definition of an outbreak; for instance, the CDC defines an outbreak of CFP as two or more cases [192], whereas the state of Florida defines an outbreak as one or more cases [193]. These issues can potentially confound the interpretation of research and surveillance data across studies. These issues suggest the potential usefulness of developing a CFP case definition that can be applied to various scientific and public health purposes, as well as across geographic regions (see case definition, Box 1).

## 7. Climate and Other Environmental Change

The geographic regions in which *Gambierdiscus* species have been identified, and CFP outbreaks reported, appear to be expanding [16]. For example, within the past 13 years, published reports indicate that *Gambierdiscus* spp. have been identified in previously un-reported CFP areas, including the western Gulf of Mexico [38,194], eastern Mediterranean [195], Crete [196], Brazil [197], Hong Kong [198], Thailand [199], and West Africa (eastern Atlantic Ocean [200,201]). In addition, there have been recent reports of CFP illness and ciguatoxic fish in previously unreported areas, such as the Canary Islands (eastern Atlantic Ocean) and the Japanese home islands [202,203]). Recent outbreaks in the United States were traced to fish from the Flower Garden Banks National Marine Sanctuary, an area in the western Gulf of Mexico previously not associated with CFP or ciguatoxic fish (FDA Advisory, FDA 2008 [204]). While the appearance of ciguatoxic fish in this region was not linked to climate change, the observed expansion in spatial distribution of both *Gambierdiscus* spp. and ciguatoxic fish has stimulated interest in better understanding the role that climate change may play in CFP.

Research has begun to identify the possible environmental predictors of CFP outbreak locations [179,205], including regional sea surface temperatures (SST), atmospheric carbon dioxide concentrations, and ocean pH [206,207].

Some studies indicate that higher *Gambierdiscus* spp. concentrations, wider latitudinal ranges, and longer growing seasons in subtropical-temperate areas [194,208,209,210] may be associated with increased ocean temperatures, and that some species of *Gambierdiscus* exhibit a wider range of tolerance than others to environmental conditions [211].

There are some indications that local SST is correlated with CFP incidence [179,205,212], although this is not a consistent finding [180,213]. It may be that the relationship between SST and CFP incidence is a function of multiple factors, including the possible suppression of some *Gambierdiscus* species when temperatures exceed certain levels [214]. In addition, methodological issues, such as the lack of historic baseline data for the *Gambierdiscus* distribution and abundance, and socio-economic factors affecting CFP reporting or fish consumption [31,180,205,214,215], limit constructive interpretation.

Carbon dioxide concentrations may also influence the abundance of CFP-associated dinoflagellates. High atmospheric carbon dioxide concentrations are associated with ocean acidification and potential shifts in biological processes such as photosynthesis, nutrient uptake, growth, reproduction, community structure, and diversity of marine biota [216,217,218,219,220,221,222,223]. Although direct evidence for the impacts of carbon dioxide concentrations is still lacking, cellular processes (such as toxin production) have been shown to be sensitive to shifts in CO_2_ concentrations [224,225,226,227], including in dinoflagellates [228,229,230,231]. A range of other climate and other environmental change-sensitive factors may affect CFP incidence, as well as other harmful algal bloom (HAB) occurrences [232]. A list of sources addressing such environmental variables is provided in Table 6.

## 8. Social Impacts

Historically, CFP has impacted regions, peoples and societies, including human migration patterns [213], local fishing practices [183,274,275], and dietary practices [276]. In Polynesia, as far back as A.D. 1000 to A.D. 1450, due to reliance on a fish-based diet, CFP may have contributed to waves of human emigration and dietary shifts away from ciguatoxic predatory fishes [213,277,278]. In recent times, a decrease in the CFP incidence was linked to increased deep water fishing practices (i.e., further from shore) where there is a reduced chance of catching ciguatoxic fish [183,274,276]. In Rarotonga, Cook Islands, in the Pacific, a study examining food consumption patterns from 1989 to 2011 revealed that the peak incidence in hospital CFP cases from 2004 to 2006 coincided with the period of highest imports of chicken into the Cook Islands and with increased consumption of canned fish [276]. It was suggested that ciguatera outbreaks, in addition to cultural factors related to modernization, may have contributed to a reduction in traditional fishing practice, disruption of the generational transmission of traditional fishing knowledge, and a culture-wide dietary shift towards a more “Westernized” or non-traditional diet.

There are also important economic impacts of CFP. In the United States, the economic impact of CFP was estimated at US$21 million annually [279] for the period from 1987 to 1992. Economic impacts have been evaluated in other world regions, such as Tahiti [280], Rarotonga in the southern Cook Islands [281], Canada [282,283], Pacific locations [183], and Puerto Rico [13]. CFP has been associated with reduced fishery productivity [183,279,280], government restrictions on the sale of potentially ciguatoxic fish [190,284,285,286,287], medical costs [281,283,288], lost worker productivity [280], reduced tourism and recreational fishing [183,279], and the costs of public health programs to monitor and manage the risk of CFP [276,289,290]. These impacts vary according to contextual factors, such as the availability of processed food, CTX prevalence, fishery dependence, social customs, and demographic trends [179,236,291,292].

## 9. International Trade, Tourism, and Traceability

The prevailing view of CFP has focused on localities where CFP is endemic. However, this focus is changing as the global demand for seafood is increasing. Currently, nearly 40% of global seafood production moves in international markets [293,294]. Recent analysis revealed that nearly 200 countries or territories were engaged in the international seafood trade, with approximately 7000 active trading partnerships. This represents a 65% increase over the last 20 years in the number of individual pathways in which seafood moves [295]. Therefore, the market dynamics affecting diagnosis and management of CFP, i.e., accurate species labeling and traceability procedures, are increasingly played out in an international setting.

In addition, international travel is a rapidly growing part of the economic system, with epidemiologic implications for tourists and other travelers [34,35,36,37,44]. For instance, two recent CFP clusters in France involved 10 people who consumed barracuda (*Sphyraena barracuda*) and grey snapper (*Lutjanus griseus*), which they had brought back from their travels in Guadalupe [33]. The United Nations World Tourism Organization estimated that there were 1.2 billion “international tourist arrivals” in 2015, which has increased by 4.4% or more every year since 2010 [296]. Thus, tourists unfamiliar with CFP are increasingly exposed to the risk [297].

Diagnosis and prevention of CFP rely on information about the species of fish consumed, and the geographic origin of the fish, which is used to inform the public and fish processors about high risk areas. Accurate species identification is also relevant to customers wishing to make informed choices to avoid CFP high risk species. However, studies suggest that internationally, comprehensive information about fish products, such as the commercial name, scientific name, and geographical source, are often not available [298], and that 25%–40% of seafood in commercial channels may be mislabeled by species [299,300,301,302,303,304]. In addition, international seafood supply chains are generally longer and more complex than those of locally or domestically traded fish. It was noted, for instance, that fish processing companies in Europe started outsourcing to China some processing steps such as filleting of certain species, in order to sell the end product back in Europe, a practice which presents increased traceability challenges [305].

Multi-national, national, and regional efforts have been established in many areas around the world to address these issues. In the European Union, for instance, there has been a coordinated multinational effort to develop [306] process-oriented controls throughout the food chain [307]. Policy reforms in the past 15 years have addressed the issue of providing traceability and production information about products from EU waters. Control measures have also been applied to imports entering the EU from non-EU countries [305,307,308,309].

In the United States, there have been a variety of policy initiatives [310], such as (1) the Bioterrorism Act of 2002, requiring that all food including fish be traceable to the source, i.e., tracked along the “chain-of-custody”, from the capture/harvest of a fish to its consumption; (2) Country-Of-Origin Labeling (COOL) requirements for wild and farm-raised seafood products under the U.S. Department of Agriculture; (3) a Presidential Memorandum entitled “Comprehensive Framework to Combat Illegal, Unreported and Unregulated Fishing and Seafood Fraud” (2014) calling upon executive departments to use existing authority to combat illegal, unreported, and unregulated fishing and fraud; (4) the U.S. FDA Fish and Fishery Products guidance, which aims to minimize potential species-related and process-related hazards in seafood (i.e., HACCP, discussed below); and (5) the U.S. FDA requirement that regardless of fish origin, acceptable market names be used for seafood sold in interstate commerce (see FDA’s Guide to Acceptable Market Names at http://www.accessdata.fda.gov/scripts/fdcc/?set=seafoodlist) [311]. Despite challenges in the implementation of international traceability and seafood labeling systems [312,313], these examples represent a growing international awareness of the complex factors contributing to risk management within the global seafood market.

## 10. Prevention and Management

Prevention and management efforts include (1) avoiding exposure to ciguatoxic fish, or fish from ciguatoxic areas; (2) surveillance and reporting of CFP to public health agencies; (3) education and outreach to consumers and professionals; and (4) poison control center support.

### 10.1. Avoiding Capture or Harvest of Ciguatoxic Fish

To reduce the likelihood of CFP, fish processors (i.e., persons engaged in the harvest, capture, handling or other aspects of fish production) are advised to not purchase or harvest fish from areas that are known to be endemic for CFP. Prohibitions against harvesting high-risk fish species have been adopted in CFP-endemic regions such as Puerto Rico [284], Cuba [285,286], the Dominican Republic [287], and in Australia [190].

Within the United States, the FDA issued the Fish and Fishery Products Hazards and Controls Guidance Fourth Edition—April 2011 (www.fda.gov/Food/GuidanceRegulation/GuidanceDocumentsRegulatoryInformation/Seafood/ucm2018426.htm) [5] that identifies CFP as a hazard reasonably likely to occur under certain circumstances, for which controls must be put in place. The FDA recommends establishing a Hazard Analysis and Critical Control Point (HACCP) Plan, whereby the primary processor will identify controls for the hazard of CFP (FDA, 2011). The FDA also issued its “Guidance for Industry: Purchasing Reef Fish Species Associated with the Hazard of Ciguatera Fish Poisoning” in 2013 (www.fda.gov/Food/GuidanceRegulation/GuidanceDocumentsRegulatoryInformation/ucm375214.htm) [314], which provides updated recommendations for primary processors to avoid endemic harvest areas for species associated with CFP. In addition, the FDA may issue new advisory notices to seafood processors, providing updated information about CFP outbreaks in regions where it was previously considered to be rare (e.g., the Flower Garden Banks of the Gulf of Mexico) [204].

In endemic fishing communities, local knowledge, outreach, and practices may help fishers to avoid ciguatoxic species or high risk CFP fishing locations [15,315], but such knowledge has limitations as a prevention strategy in the absence of adequate CFP incidence data or relevant *Gambierdiscus* data for such locations.

### 10.2. Surveillance and Reporting

Within the United States, public health officials voluntarily report outbreaks of CFP to the Centers for Disease Control and Prevention’s (CDC) surveillance system, the National Outbreak Reporting System (NORS) (www.cdc.gov/nors/index.html) [316]. CDC publishes Annual Summaries of Foodborne Outbreaks (www.cdc.gov/foodsafety/fdoss/data/annual-summaries/index.html) [317], including CFP. In addition, a new module for NORS, One Health Harmful Algal Bloom System (OHHABS; launched Summer 2016 www.cdc.gov/habs/ohhabs.html) [318], allows public health officials to enter single cases of CFP in addition to outbreaks. OHHABS is designed to capture data relevant to CFP events, including case characteristics and environmental data; and over time, it may provide data to be used to improve CFP diagnosis and treatment as well as CFP epidemiology and surveillance.

In addition, within various U.S. states and territories (e.g., Florida, Hawaii, Rhode Island, U.S. Virgin Islands, North Carolina, New York, South Carolina, and California) CFP is a “reportable” or “notifiable” condition (i.e., required to be reported to public health officials).

In Florida where CFP is endemic, multiple interlocking reporting systems have been implemented to improve detection and response to CFP outbreaks [189,193]. Cases can be detected via (1) county health departments which make reports to state health authorities through an electronic surveillance system; (2) hospital emergency departments; (3) Florida Department of Health’s online food complaint form (http://www.Floridahealth.gov/diseases-and-conditions/food-and-waterborne-disease/online-food-complaint-form.html) [319] which allows self-reports by affected individuals; and/or (4) calls to the poison control hotline (1-800-222-1222) which are answered by the Florida Poison Information Centers (www.Floridapoisoncontrol.org) [320].

In French Polynesia, where CFP is also endemic, it is an officially notifiable disease as part of a government surveillance program supervised by the Public Health Directorate, although notification by private physicians is optional [15].

Reporting of potential cases to local or state health departments enables public health authorities to assist in the investigation of potential cases, identify outbreaks, and help prevent further CTX exposures. Within the United States, local and state health departments may collaborate with the FDA to facilitate analytical testing on a remnant of the suspect fish, and if indicated, fish trace-back procedures, inspections, and regulatory actions to prevent additional exposures. The FDA then uses data obtained from its fish analysis procedure, regarding the identified species, toxin concentrations, and fish origin, to refine guidance to the seafood industry as appropriate.

### 10.3. Education and Outreach

Educating the public and healthcare providers about CFP is a critical component of CFP prevention [188,321,322,323], as it improves the ability to diagnose and treat cases of CFP quickly and enables consumers to make informed choices about what to eat.

Targeted educational messages, to inform the public, fishers, fish vendors, and restaurants about where to obtain good information on CFP, help to raise awareness. Information can be obtained online at a number of reputable sources, including the CDC’s ciguatera web page (www.cdc.gov/nceh/ciguatera/default.htm) [324] which also provides information about fish that cause CFP (www.cdc.gov/nceh/ciguatera/fish.htm) [325], the FDA’s *Bad Bug Book* (www.fda.gov/Food/FoodborneIllnessContaminants/CausesOfIllnessBadBugBook/default.htm) [51] and *Fish and Fishery Products Hazards and Controls Guidance* published by the FDA [326].

States within the United States have created public alerts or information sheets detailing symptoms or identifying implicated fish. Examples include this Hawaii information sheet (http://health.Hawaii.gov/docd/files/2013/05/dib_ciguatera.pdf ) [327] and this Florida ciguatera-facts flyer (http://www.Floridahealth.gov/environmental-health/aquatic-toxins/ciguatera-facts-card-2015.pdf) [328], each provided by their respective state department of health website.

Culturally-tailored education efforts are indicated for specific at-risk populations, such as certain Hispanic residents of Miami-Dade County, Florida, who traditionally consume barracuda [182]. In addition, posters aimed at local fishers regarding which locations are associated with known ciguatoxic fish may help to reduce CFP occurrences.

In French Polynesia, the Ciguatera-Online website of the Institut Louis Malardé (www.ciguatera-online.com) [329] provides a wide range of information on CFP. The Institut Louis Malardé is an organization dedicated to research and surveillance on CFP in French Polynesia, and conducts research to develop tools to detect *Gambierdiscus*, epidemiological monitoring of CFP, risk mapping of sensitive Polynesian lagoons, environmental mechanisms underlying toxic algal blooms, assessment of traditional medicines, biomedical studies, and health monitoring. The website provides downloadable information and documents, examples of CFP educational brochures and posters in multiple languages, CFP interactive maps, statistics on CFP epidemiology in French Polynesian islands including ciguatoxic fish species involved in CFP outbreaks, and other informational links pertaining to CFP. It also has mechanisms for reporting CFP cases as well as potentially toxic fishing locations.

For outreach efforts to be helpful for residents, they must be accessible, acknowledge local beliefs, and connect people to trusted resources for immediate diagnosis, reporting and treatment. Evaluations of outreach methods and interventions thus far are rare, but will be key to learning if they are effective in reducing the incidence of CFP over the long term [330].

### 10.4. Assistance from Poison Control Centers

Assistance for healthcare providers or patients who suspect a diagnosis of CFP is available from local poison control centers world-wide, a directory of which is provided by the World Health Organization at http://www.who.int/gho/phe/chemical_safety/poisons_centres/en/ [331]. For example, within the United States, Canada, and U.S. territories in the Caribbean, there is a network of 55 poison control centers that can be accessed 24 h a day by calling 1-800-222-1222 (outside the U.S. +011-800-222-1222). Poison control specialists are doctors, nurses, or pharmacists with extensive training managing poisonings, including foodborne illness (www.aapcc.org). Physician consultation with a medical toxicologist is also available free of charge. In populations familiar with the illness, there may be a need to promote seeking treatment, particularly within the first 72 h [182], and official reporting of the illness. Many people (including healthcare providers) may not realize that early treatment may minimize the severity and duration of symptoms. Encouraging interactions with local poison control centers may facilitate more accurate diagnosis, treatment, and tracking of CFP.

## 11. Future Directions

CFP stands at the international crossroads of epidemiology, ecology, technology, culture, and commerce. This review reveals a number of opportunities to advance the field across multiple disciplines.

Worldwide, there is a need for improved surveillance of CFP to obtain baseline as well as longitudinal epidemiological data across geographic regions. This information should be collected in parallel with the ecological surveillance of *Gambierdiscus* species, presence and abundance, to identify environmental predictors of CFP worldwide [182,185,205,232,323,332,333]. Collaborative, multi-agency, systematic risk assessment frameworks [334] are desirable to collect and share environmental and public health data and ultimately to reduce exposures to CTXs [333].

Fundamental to the improvement in surveillance is accurate diagnosis and reporting. To this end, the education of health professionals potentially involved in CFP diagnosis and reporting, and of specific at-risk groups (e.g., fishers), is recommended.

In addition, there continues to be a need for the development of simple, cost-effective methods of detecting CTXs in fish, which can be used by fishers, vendors, and consumers, and can provide satisfactory levels of accuracy [176].

The development of methods to confirm exposure to CTX in affected humans will assist not only in the diagnostic process, but also in the validity of clinical trials investigating treatments for individuals with a presumed CFP diagnosis. In the effort to develop biomarkers, progress has been made particularly in animal studies [112,113,114,115,116]. In humans, attempts have been made to identify CTX in human blood [335] and other potential markers of effect of CTX exposure [336,337,338], but studies are needed with larger sample sizes, clearly delineated case definitions, confirmatory fish testing across laboratories with appropriate blinding, and replication of candidate biomarkers across multiple study populations.

The physiological mechanisms of CTX during the acute phase of the illness raise questions about the evolution of CTX effects over time. It has been shown that mannitol reduces cellular edema by preventing the influx of sodium and water into the cell. However, it appears that mannitol may not exhibit this benefit beyond 72 h after toxin exposure; this suggests that at such point, the sodium channel receptors may have undergone changes or even damage due to the CTX exposure. Animal studies may provide the opportunity to examine the effects of CTX on neural membranes, acutely and over time.

There is a need for prospective epidemiologic studies to clarify the longevity of CFP symptomatology in humans and the nature and mechanisms of the reported symptom recurrence, sensitization, or cross-sensitization.

Randomized clinical trials of sufficient size with well-defined protocols and confirmed cases of CFP are needed to document the efficacy of IV mannitol and other common treatments. Future developments may include the ability to produce effective and safe CTX antibodies suitable for human treatment [339].

Continued development of methodologies for tagging and tracking the geographic origin (including latitude and longitude), size and species of fish, in an ever-expanding international market, are desirable to improve the accuracy of epidemiological data about CFP vectors and their geographic origins. DNA barcodes [156,157] and Radio Frequency Identification Device (RFID) tags have been used for authentication and tracking of seafood in commerce [298,305,313,340,341,342]. However, there is a need for rapid handheld instruments that can be used by fishers, wholesalers, and retailers. Tracking and authentication technologies, commodity tracking systems, and the development of regulatory protocols/agreements show promise that the seafood market system can be traceable and accurately assessed in the future [340,343].

A more systematic approach to acquiring field data is needed to understand the social, economic, and cultural impacts of CFP. Development of standardized methods of measuring the impacts would allow improved future prediction of impacts. Metrics should, where feasible, include estimating the economic burdens of CFP across the range of possible impacts (e.g., health, economic, cultural) to assess the need for CFP prevention and treatment programs, particularly in developing nations.

International multidisciplinary collaborations are desirable. An example is the GlobalHAB program funded by the National Science Foundation (through the Scientific Committee on Oceanic Research) and the Intergovernmental Oceanographic Commission of the United Nations Educational, Scientific and Cultural Organization (UNESCO). This program aims to foster coordinated multidisciplinary research on HABs, including CFP. GlobalHAB focuses on the human health impacts of marine microalgae-produced toxins, the fundamental ecophysiology and oceanic processes that modulate HAB dynamics, and on the intersection of ecology and epidemiology of HAB illnesses [344]. As another example, a “Global Burden of Disease” CFP study (e.g., http://www.healthdata.org/gbd [345]) would allow for a clearer understanding of the current extent and costs and other impacts from CFP, as well as future changes in CFP distribution in the context of international economic, cultural, and environmental change. Finally, the One Health approach (see http://onehealthinitiative.com/ [346]) intrinsically includes cooperation across environmental and health disciplines and could be used effectively to address CFP diagnosis, treatment, and reporting worldwide.

**Ciguatera Fish Poisoning: Information for Clinicians**

Based upon available evidence and anecdotal experience, the authors recommend the following for CFP clinicians. Some of these recommendations are specific to public health management procedures within the United States; various other countries and jurisdictions have developed their own respective procedures.

1. **Diagnose early**: Consider the diagnosis of CFP in the context of any seafood-borne illness, rule out other possible diagnoses, and explore whether people other than the initial case have been exposed. Patients diagnosed with CFP without fish testing confirmation should be warned about the uncertainty of a purely clinical diagnosis and recommended for additional medical evaluation if symptoms recur or do not resolve.
2. **Obtain and submit suspect fish sample for CTX analysis**: Within the United States, if a suspect case of CFP has been identified, the implicated fish meal remnants and the case-related information may be sent to the FDA for CTX analysis. For an FDA consultation and instructions on submitting meal remnants, contact Ronald Benner (Ronald.Benner@fda.hhs.gov), phone: (251) 406-8124.
3. **Report**: Even a single case of possible CFP should be reported to state or local health authorities as soon as possible. Within the United States, in some states (e.g., Florida), licensed physicians and certain other practitioners are required by law to report suspected cases. Within the United States, assistance in reporting can be obtained by contacting your local poison control center by calling 1-800-222-1222, 24 h/day.
4. **Use of intravenous (IV) mannitol**: If a patient is diagnosed with CFP, consumed the implicated fish within the past 48–72 h, and there are no contra-indications to its use, then treatment with IV mannitol is recommended. After 72 h, IV mannitol treatment may be considered on a case by case basis. Prior to treatment, patients should be informed about the inconsistency of research findings on the effectiveness of IV mannitol for CFP, the limitations of knowledge about its effects, that it may not work, that insurance may not pay for this treatment, and that subsequent treatments might be recommended if it does work, as symptoms may return after initial successful treatment. In all cases, the decision to proceed with mannitol treatment should be based upon the risk–benefit analysis, discussing such risks and benefits with the patient, and ultimately, the preference of the patient upon being provided with such information.
5. **Supportive and symptomatic treatments**: Supportive and symptomatic medical treatments for specific CFP symptoms should be determined on a case by case basis, according to the patient health situation. Caveat: there are no randomized controlled studies investigating the effectiveness of any medical treatments other than mannitol for CFP. Caution is warranted in prescribing medications with addictive potential.
6. **For patients with more lasting complaints that are *not* clearly caused by CFP**, it is recommended that they receive a full evaluation by a neurologist, internist, psychologist, and/or psychiatrist, who can provide joint input on the diagnosis and a recommended plan of care.
7. **For patients with more lasting complaints that *do* appear to be caused by CFP**, patients may benefit from a low dose of selective serotonin reuptake inhibitor, as well as a combination of assessment and care including medical follow-up, physical therapy, and psychotherapy interventions such as cognitive behavioral therapy, biofeedback, or family counseling sessions, as needed, on a case by case basis.
8. **Fish avoidance**: Consumers may choose to avoid consuming fish associated with CFP, from regions associated with CFP, large reef fish, large fish portions, and fish head and organs.
9. **Avoiding symptom recurrence**: It is recommended that patients avoid becoming dehydrated, and avoid consuming alcohol, nuts, caffeine, pork, chicken, and any type of fish, for 3–6 months after CFP intoxication or until symptom-free. Alternatively, patients may opt to try certain items, with caution, watching for recurrent symptoms. This recommendation is made with these caveats: (a) this is based on anecdotal reports, not empirical studies; and (b) this avoidance recommendation may not be helpful to all CFP patients.
10. **Education**: Within hospital emergency departments, poison control centers, public health departments, and medical school programs, educational modules for healthcare staff and students should be implemented.

This information text box from: Friedman, et al., Marine Drugs 2017. DOI:10.3390/md15030072.

**Ciguatera Fish Poisoning: Information for Patients**

**1.**  **What is ciguatera fish poisoning (CFP)?** CFP is a food poisoning illness caused by eating finfish contaminated with naturally-occurring toxins called “ciguatoxins”.
**2.**  **What fish types cause CFP?** CFP is usually caused by eating certain species of finfish, usually those which live or feed near coral reefs.
**3.**  **What are the symptoms?** Patients typically experience symptoms within 6–12 h of eating the fish, and symptoms can include diarrhea, nausea, and/or vomiting, as well as tingling or burning sensations in the hands, feet, and/or mouth, muscle weakness, and fatigue. Some patients also experience circulatory symptoms such as slow heartbeat or low blood pressure.
**4.**  **What should patients do?** If you think you have CFP, go to your nearest emergency room right away. One reason to seek care quickly is that treatments for CFP (i.e., IV mannitol) may be less effective if started more than 72 h after eating the fish. In some places, such as within the United States, if hospital staff do not know about CFP (which may occur in areas where CFP is rare), the patient or their doctor can request a free consultation with a poison control center (1-800-222-1222 in the U.S), to help with diagnosis and treatment.
**5.**  **What about leftover fish?** Patients should bring leftover fish from the meal they ate with them to the emergency room, if possible. The leftovers may be sent for special testing to determine the presence of ciguatera toxins.
**6.**  **Did multiple people share the fish?** Please notify any others who ate the fish that you are sick, and ask them if they are having similar symptoms.
**7.**  **If you have not eaten fish that might contain ciguatoxins within the last 72 h, but you still think you may have CFP**, call your healthcare provider and report your concerns.
**8.**  **How does my doctor know if I have CFP or something else?** A complete medical workup for CFP may include talking with a neurologist, internist, and possibly other specialists to help rule out other conditions with similar symptoms. This is an important step because other conditions may be more easily treated than CFP.
**9.**  **Are there treatments?** Intravenous (IV) mannitol (1 g/kg) is recommended for people with CFP, especially within the first 72 h after eating the fish. Not all healthcare providers are aware of this treatment, since CFP is rare. Note: (1) Medical insurance may not pay for this treatment; and (2) even if you feel better after IV mannitol treatment, you may need to repeat it if symptoms come back.
**10.**  **If I am diagnosed with CFP, what can I do to reduce symptoms and prevent possible relapse of symptoms?** It varies from person to person, but the following may be helpful:
  - Avoid eating fish (salt water and fresh water) until you feel completely well.
  - Avoid alcohol, caffeine nuts, pork, chicken, and any type of fish for at least the first 3–6 months after intoxication.
  - Do not become dehydrated.
  - Your doctor may prescribe medications to relieve your symptoms.
  - Your doctor may refer you to other medical specialists.
  - Remember, most people recover completely from CFP; it just takes time.
**11.**  If you have been diagnosed with CFP, ask your doctor to report your case to the local public health authority so they can record your symptoms. They may need to follow up with you for additional information. This is especially important if other people got sick or if the toxic fish may still be available for people to eat.
**12.**  For more information about ciguatera fish poisoning, go to this website: http://www.cdc.gov/nceh/ciguatera/.

This information text box from: Friedman, et al., Marine Drugs 2017. DOI:10.3390/md15030072.

## Figures and Tables

**Table 1:** Examples of Common Ciguatoxic Fish Species [5] (Common name of the family in English (*and Latin*); also, some genera (g.) are cited as examples.)

Moray eel (*Muraenidae*)
Barracuda (Sphyraenidae)
Grouper (Serranidae)
Jacks (Carangidae)
Amberjack (Carangidae, g. *Seriola*)
Snapper (Lutjanidae)
Surgeon fish (Acanthuridae)
Parrot fish (Scaridae)
Wrasses (Labridae)
Hogfish (Labridae, g. *Lachnolaimus*)
Narrow barred mackerel (Scombridae, g. *Scomberomorus*)
Spanish mackerel (Scombridae, g. *Scomberomorus*)
Trevally (Carangidae, g. *Caranx*)
Triggerfish (Balistidae)

Note: Various other examples of ciguatoxic fish are reported around the world [6,7,8,9,10,11,12,13,14,15].

**Table 2:** Reported Frequency (%) of Clinical Symptoms of Ciguatera Fish Poisoning (CFP) at Time of Diagnosis ^1^.

Ocean Region:	CARIBBEAN						ATLANTIC		PACIFIC							INDIAN
First Author and Year:	Friedman 2007 [65]	Arena 2004 [64]	Stinn 2000 [48]	Frenette 1988 [73]	Engleberg 1983 [74]	Escalona 1985 [67]	Lawrence 1980 [75]	Baumann 2010 [76]	Gatti 2008 [77]	Chateau-Degat 2007 [71]	Chateau-Degat 2007 [78]	Schnorf 2002 [79]	Bagnis 1987 [80]	Gillespie 1986 [6]	Bagnis 1979 [54]	Quod 1996 [62]
*Number of Study Participants (N)*	*N* = 12	*N* = 12	*N* = 442	*N* = 57	*N* = 47	*N* = 80	*N* = 129	*N* = 210	*N* = 124	*N* = 1824	*N* = 47	*N* = 50	*N* = 12,890	*N* = 527	*N* = 3009	*N* = 167
*Gastrointestinal:*																
Diarrhea	67	75	79	77	81	83	76	44	80	77		50	73	64	71	49
Vomiting		42	43	37	40	69	68	28	55	32			39	35	38	50
Nausea	42			82		69			17			26	44	55	43	50
Abdominal Pain	42	75	65	58	30	74			40			52	43	52	46	29
*Neurologic:*																
*Peripheral Nervous System Symptoms:*																
Paresthesia-Extremity	67	100	81	79		36	71	95	49	89 ^2^	93	72	89	64–41	89	82
Temperature Dysesthesia	58	92	64	77	23	48		81 ^3^	16 ^4^	89 ^5^	34	94	87	76	88	65
Circumoral Paresthesia	58		70	79	38	38	54		31		91		88	66	89	82
Dental Pain/Feeling Like Teeth Are Loose	33		32	23	13	11		6					21	37	25	
Myalgia	67	75	79	75	34	56	86	84 ^6^	12	84 ^7^	80	56	85	83	82	38
Arthralgia	42	83	79	75	34	60		84 ^8^	6		80	62	86	79	86	29
Pruritis	67	67	77		66	45	48	60	64			42	44	76	45	5
Dysuria	8	33	25					5	1.6	23		26	13	22	19	
*Central Nervous System Symptoms:* ^9^																
Vertigo/Dizzy/Lipothymy	25	58	50			33	47		31	56		62		45	42	
Loss of Consciousness									10 ^10^							
Cerebellar Syndrome									11							
Balance Disturbance								27								
Hallucinations	8	17													<5	16
Depression	25	17														16
Memory/Concentration	17	58														
Behavioral Disturbance									4							
Visual Disturbance								9	3							
Multi-Tasking Problems	25															
Giddiness													29			30
*Cardiovascular:*																
Bradycardia	3							8 ^11^	75	13		16	16			
Hypotension								15	43	8						
Hypertension									5			12	12			
Tachycardia								8 ^12^	5	1						
Arrhythmia	33															
*Other: *																
Headache				56	45	39	47	27	9	51		50	60	62	59	25
Weakness/Asthenia/Fatigue	92	100		84		65	30	89	34			80	60		60	70
Respiratory Disturbance								7	5							
Chills/Sweating					**36**		**24**	**39**	**3** ^13^				**60**	**49**	**59**	

Notes: Blank cells indicate that data on that symptom were not reported in the study referenced. The table does not provide relative risk data, i.e., it does not provide comparative information on symptom frequency in an unexposed population. The table is modified from Stinn et al., 2000 [48]; Arena et al., 2004 [64], and Friedman et al., 2008 [1]. ^1^ Not all symptoms from all studies are included here. The most consistently reported, and high frequency symptoms, are included; ^2^ Chateau-Degat et al. (2007) [71] report a single variable “Paresthesia”, with a frequency of 89%; ^3^ Baumann et al. (2010) [76] also report on a separate variable, “Disturbance of Sensation on Contact with Water” with a frequency of 90%; ^4^ Gatti et al. (2007) [77] refer to this variable as “Dysesthesia”, without specific regard to temperature; ^5^ Chateau-Degat et al. (2007) [71] refer to this variable as, “Trouble with cold perception” with a frequency of 89%; ^6^ Baumann et al. (2010) [76] refer to this symptom as muscle/joint aches, with a reported symptom frequency of 84%; ^7^ Chateau-Degat et al. (2007) [71] report a single variable “Pain”, with a frequency of 84%; ^8^ Baumann et al. (2010) [76] refer to this symptom as muscle/joint aches, with a reported symptom frequency of 84%; ^9^ In addition to the symptoms reported in this table, Gatti et al. (2007) [77] reported a small percentage of patients with symptoms that may be reflective of central nervous system effects, such as diplopia (0.8%), dysguesia (0.8%), language disturbance (0.8%); ^10^ Gatti et al. (2007) [77] reported, in addition, that 4% experienced a disturbance of consciousness; ^11^ Baumann et al. (2010) [76] list the symptoms as tachy- or bradycardia, with a reported frequency of 8%; ^12^ Baumann et al. (2010) [76] list the symptoms as tachy- or bradycardia, with a reported frequency of 8%; ^13^ Gatti et al. (2007) [77] report this variable as “shivering”.

**Table 3:** Examples of some illnesses with clinical presentations similar to Ciguatera Fish Poisoning (CFP).

Illness	Cause	Symptom Presentation
Paralytic Shellfish Poisoning	Caused by ingestion of marine bivalve mollusks such as mussels, clams, and oysters, contaminated with saxitoxins	Within minutes of ingestion, there is onset of intraoral and perioral paresthesia, particularly of the tongue and gums similar to ciguatera but slower in onset. Paresthesias are rapidly followed by weakness, dysarthria, dysphagia, and other symptoms. The mortality rate is estimated at 25%, or higher in children [98,99].
Pufferfish (Fugu) Poisoning	Caused by ingestion of pufferfish contaminated with tetrodotoxins	Paresthesia of the face and extremities, nausea, dizziness, loss of reflexes, weakness and paralysis. The marked weakness and paralysis of pufferfish poisoning is not seen in CFP.
Neurotoxic Shellfish Poisoning	Caused by ingestion of molluscan shellfish contaminated with brevetoxins	Nausea and vomiting, paresthesias of the mouth, lips, tongue, and extremities, ataxia, slurred speech, and dizziness. Neurologic symptoms can progress to partial paralysis; respiratory distress may occur.
Scombrotoxin Fish Poisoning	Caused by ingestion of fresh, canned or smoked fish with high histamine levels due to improper processing or storage	Flushing, rash, hives, palpitations, headache, dizziness, sweating, and burning of the mouth and throat; abdominal cramps, nausea, vomiting and diarrhea; bronchospasm, respiratory distress and vasodilatory shock may occur.
Botulism	Caused by ingestion of canned foods contaminated with botulinum toxin.	Vomiting, diarrhea, abdominal pain, extraocular muscle weakness, dysphagia and respiratory paralysis and unless promptly treated in the intensive care setting may result in death. Unlike in CFP, there are no sensory symptoms.
Guillain–Barré Syndrome	Cause unknown. Believed to be an autoimmune reaction in response to a viral or bacterial infection.	Acute inflammatory demyelinating polyradiculoneuropathy, which may present with paresthesia followed by weakness of the extremities, loss of reflexes and in severe cases dysphagia and respiratory failure. The early onset paresthesia may resemble ciguatera, especially if there is a history of gastrointestinal symptoms prior to the onset of paresthesia and history of having eaten fish associated with CFP.
Acute Arsenic poisoning	Caused by the intentional or unintentional ingestion of arsenic.	May present with gastrointestinal symptoms and subsequent peripheral neuropathy. Unless there is suspicion of arsenic ingestion, the diagnosis of arsenical neuropathy may be overlooked, and CFP may be wrongly diagnosed.
Organophosphate poisoning	Caused by dermal, inhalational or oral exposure to organophosphate compounds, usually pesticides.	Initial symptoms of vomiting, diarrhea and abdominal pain may resemble CFP. It may cause delayed sensory and motor peripheral neuropathy. Unlike CFP, it has cholinergic symptoms of salivation, bronchorrhea and bronchospasm.
Acute bacterial or viral gastroenteritis	Caused by ingestion of contaminated food or exposure to infectious individuals	Nausea, vomiting and diarrhea alone or combined, with or without neurologic symptoms (enterotoxigenic *E. coli*, Shigella) in patients with a history of exposure.

**Table 4:** Foods and behaviors associated with Ciguatera Fish Poisoning (CFP) symptom recurrence.

Alcohol [6,34,81,88,108]
Nuts [88,108]
Caffeine [88,108]
Pork [108]
Chicken [6,88,108]
Any fish, including freshwater [6,81,88,108]
Physical activity/exertion/dehydration [34]

**Table 5:** Published incidence estimates of CFP per 10,000 population in select locations.

Location	Time	Estimated Incidence Rate	Reference
***Caribbean***			
US Virgin Islands (St. Thomas ER records)	1970–1979	180 (adults)	Radke et al., 2013 [180]
Illes Santes (Guadaloupe)	1960–1980	30	Czernichow et al., 1984 [181]
Florida (Miami)	1974–1976	≥5	Lawrence et al., 1980 [75]
Montserrat	1996–2006	58.6	Tester et al., 2010 [179]
Antigua and Barbuda	1996–2006	34.4	Tester et al., 2010 [179]
British Virgin Islands	1996–2006	19.9	Tester et al., 2010 [179]
Bahamas	1996–2006	5.8	Tester et al., 2010 [179]
Cayman Islands	1996–2006	2.9	Tester et al., 2010 [179]
US Virgin Islands (St. Croix)	1996–2006	2.3	Tester et al., 2010 [179]
Aruba	1996–2006	1.6	Tester et al., 2010 [179]
Grenada	1996–2006	0.6	Tester et al., 2010 [179]
Guadeloupe	1996–2006	0.3	Tester et al., 2010 [179]
Martinique	1996–2006	0.2	Tester et al., 2010 [179]
Dominican Republic	1996–2006	0.05	Tester et al., 2010 [179]
Jamaica	1996–2006	0.04	Tester et al., 2010 [179]
Puerto Rico	1996–2006	0.03	Tester et al., 2010 [179]
Colombia	1996–2006	0.003	Tester et al., 2010 [179]
Puerto Rico (Culebra)	2004–2006	40 ^a^ 75 ^b^	Azziz-Baumgartner et al., 2012 [13]
Florida (all)	2007–2011	0.56 ^c^	Radke et al., 2015 [182]
Florida (Miami-Dade)	2007–2011	2.8 ^c^	Radke et al., 2015 [182]
Florida (Monroe)	2007–2011	8.4 ^c^	Radke et al., 2015 [182]
Florida (fishers)	2011	1.7 ^d^	Radke et al., 2015 [182]
US Virgin Islands (St. Thomas phone survey)	2010–2011	120	Radke et al., 2013 [180]
US Virgin Islands (St. Thomas ER records)	2007–2011	60 (adults)	Radke et al., 2013 [180]
***Pacific***			
American Samoa	1973–1983	8.7	Lewis 1986 [183]
Cook Islands	1973–1983	0.1	Lewis 1986 [183]
Fiji	1973–1983	1.6	Lewis 1986 [183]
French Polynesia	1973–1983	54.5	Lewis 1986 [183]
Guam	1973–1983	0.8	Lewis 1986 [183]
Kiribati	1973–1983	32.4	Lewis 1986 [183]
Nauru	1973–1983	0.7	Lewis 1986 [183]
New Caledonia	1973–1983	20.0	Lewis 1986 [183]
Niue	1973–1983	13.0	Lewis 1986 [183]
Papua New Guinea	1973–1983	>0.1	Lewis 1986 [183]
Solomon Islands	1973–1983	0.2	Lewis 1986 [183]
Tokelau	1973–1983	65.3	Lewis 1986 [183]
Tonga	1973–1983	2.1	Lewis 1986 [183]
TIPI	1973–1983	17.3	Lewis 1986 [183]
Tuvalu	1973–1983	43.9	Lewis 1986 [183]
Venuatu	1973–1983	2.5	Lewis 1986 [183]
Wallis and Futuna	1973–1983	0.9	Lewis 1986 [183]
Western Samoa	1973–1983	5.4	Lewis 1986 [183]
Federated States of Micronesia	1982–1983	0.2	Lewis 1986 [183]
Marshall Islands	1982–1983	28.2	Lewis 1986 [183]
Commonwealth of the Northern Marianas	1982–1983	13.0	Lewis 1986 [183]
Palau	1982–1983	0.0	Lewis 1986 [183]
Hawaii	1975–1981	20.3	Anderson et al., 1983 [184]
French Polynesia (all)	1992–2001	36 ^e^ 36 ^f^	Chateau-Degat 2007 [71]
French Polynesia (Austral)	1992–2001	197 ^e^ 193 ^f^	Chateau-Degat 2007 [71]
French Polynesia (Marquesas)	1992–2001	251 ^e^ 280 ^f^	Chateau-Degat 2007 [71]
French Polynesia (Tuamotu)	1992–2001	165 ^e^ 156 ^f^	Chateau-Degat 2007 [71]
French Polynesia (Society)	1992–2001	10 ^e^ 10 ^f^	Chateau-Degat 2007 [71]
***Elsewhere***			
Australia (Cairns and Maryborough)	1984	3.0	Gillespie et al., 1986 [6]
Réunion Island (Indian Ocean)	1986–1994	0.78	Quod and Turquet 1996 [62]
Hong Kong	1989–2008	0.102 (median) 0.649 (peak 1998)	Chan 2015 [185]
Japan (Okinawa Prefecture)	1997–2006	0.077	Chan 2015, citing Oshiro et al., 2010 [185]
China (Guangdong Province—Shenzen)	2004	0.075	Chan 2015 [185]
China (Guangdong Province—Foshan)	2004	>0.487	Chan 2015 [185]
China (Guangdong Province—Zhongshan)	2004	>1.299	Chan 2015 [185]
Japan (Okinawa Prefecture)	2004	0.065	Chan 2015, citing Oshiro et al., 2009 [185]
China (Guangdong Province—Shenzen)	2005–2006	0.011	Chan 2015 [185]
Japan (Kakeroma Island)	2005–2006	0.002	Chan 2015, citing Oshiro et al., 2011 [185]
Japan (Kakeroma Island)	2005–2008	0.22	Chan 2015, citing Oshiro et al., 2011 [185]

^a^ “possible” ciguatera; ^b^ “probable” ciguatera; ^c^ adjusted for under-reporting; ^d^ adjusted for non-response ×10; ^e^ crude; ^f^ age-standardized. Adapted from Friedman et al., 2008 [1], Tester et al., 2010 [179].

**Table 6:** Studies of select environmental change-sensitive factors potentially affecting *Gambierdiscus* spp. growth, abundances, and diversity.

Factor	Study Type	Study
Algal extracts	Laboratory	Carlson, 1984 [233]
Coral extract	Field, Laboratory	Holmes et al., 1990 [234]
Depth, Precipitation	Field, Laboratory	Carlson, 1984 [233]
Depth, Precipitation	Field, laboratory	Carlson and Tindall, 1985 [235]
Depth, Water motion	Field	Richlen and Lobel, 2011 [236]
Depth ^a^	Field	Tester et al., 2013 [194]
Environmental disturbance	Field	Kaly and Jones, 1994 [237]
Environmental disturbance, Coral bleaching	Time series	Skinner et al., 2011 [31]
Growth substances	Laboratory	Asuncion et al., 1995 [238]
Habitat type	Field	Richlen and Lobel, 2011 [236]
Habitat type	Field	Tan et al., 2013 [239]
Habitat type	Field	Yasumoto et al., 1979 [240]
Habitat type	Field	Yasumoto et al., 1980 [241]
Habitat type, Substrate Preference	Field	Ballantine et al., 1985 [242]
Habitat type, Substrate, Algal exudate	Field, Laboratory	Grzebyk et al., 1994 [243]
Herbivore grazing	Field	Loeffler et al., 2015 [244]
Interspecific toxicity	Laboratory	Rhodes et al., 2014 [245]
Latitude ^b^	Field	Nishimura et al., 2013 [203]
Nitrogen vs Toxicity	Laboratory	Lartigue et al., 2009 [246]
Nitrogen, Phosphorus, Silicon	Field	Inoue et al., 1990 [247]
Nutrients, Depth	Field	Loeffler et al., 2015 [244]
Site factors, hydrographic parameters	Field	Okolodkov et al., 2014 [248]
Site factors, Substrate	Field	Popowski et al., 2001 [249]
Substrate	Field	Kohler and Kohler, 1992 [250]
Substrate	Laboratory	Nakahara et al., 1996 [251]
Substrate preference	Field	Lobel et al., 1988 [252]
Substrate preference	Laboratory	Parsons et al., 2011 [4]
Substrate Preference, Site factors ^c^	Field	Tester et al., 2014 [253]
Substrate, Algal extract	Field, Laboratory	Bomber et al., 1989 [254]
Substrate, Runoff, Habitat type, Site factors	Field	Taylor, 1985 [255,256]
Substrate, Runoff, Habitat type, Site factors	Field	Taylor and Gustavson, 1985
Substrate, shoreline location	Field	Delgado et al., 2006 [257]
Substrate, Site factors	Field	Morton and Faust, 1997 [258]
Substrate, Site factors, Habitat type	Field, Laboratory	Carlson, 1984 [233]
Substrate, Site factors, Habitat type	Field, Laboratory	Carlson and Tindall, 1985 [235]
Substrate, Site factors, hydrographic parameters	Field	Okolodkov et al., 2007 [259]
Substrate, Site factors, nutrients, hydrographic parameters	Field	Parsons and Preskitt, 2007 [260]
Substrate ^d^	Field	Villareal and Morton, 2002 [261]
Temperature	Laboratory, Field	Adachi et al., 2012 [262]
Temperature	Time series	Chinain et al., 1999 [263]
Temperature	Time series	Hales et al., 1999 [212]
Temperature	Time series	Llewellyn et al., 2010 [214]
Temperature vs. CFP	Field, Time series	Chateau-Degat et al., 2005 [264]
Temperature, Light	Laboratory	Ballantine et al., 1992 [265]
Temperature, Rainfall	Field, Time series	Tosteson et al., 1988 [266]
Temperature, Rainfall, Toxicity	Field, Time series	Ballantine et al., 1988 [267]
Temperature, Salinity	Laboratory	Tawong et al., 2016 [268]
Temperature, Salinity, Light	Laboratory	Bomber et al., 1988 [269]
Temperature, Salinity, Light	Laboratory	Kibler et al., 2012 [270]
Temperature, Salinity, Light	Laboratory	Kibler et al., 2015 [210]
Temperature, Salinity, Light	Laboratory	Morton et al., 1992 [271]
Temperature, Salinity, Light	Laboratory	Xu et al., 2016 [211]
Temperature, Salinity, Light, Nutrients	Modeling	Parsons et al., 2010 [272]
Temperature, Salinity, Nutrients	Laboratory	Withers, 1981 [273]

^a^ Species diversity was examined at different depth ranges; ^b^ Species diversity over a range of sites in different latitudes was examined; ^c^ *Gambierdiscus* abundance on an artificial substrate vs. macroalgae, sample size, site effects; ^d^ Effect of algal substrate on photosynthesis.
